# Evaluation of changes of anterior segment parameters in patients with pseudoexfoliation syndrome after cataract surgery using anterior segment optical coherence tomography

**DOI:** 10.1038/s41598-024-58564-z

**Published:** 2024-04-09

**Authors:** Aleksandra Wlaź, Agnieszka Kustra, Tin Aung, Tomasz Żarnowski

**Affiliations:** 1 Department of Diagnostics and Microsurgery of Glaucoma, Medical University, Chmielna 1, 20-079 Lublin, Poland; 2 Optegra Eye Clinic, Gęsia 5, 20-719 Lublin, Poland; 3 Singapore Eye Research Institute & Singapore National Eye Centre, Singapore, Singapore; 4 Duke-NUS Medical School, National University of Singapore, Singapore, Singapore

**Keywords:** Pseudoexfoliation syndrome, Anterior segment OCT, Cataract surgery, Phacoemulsification, Lens diseases, Eye diseases, Eye abnormalities

## Abstract

The aim of the study was to compare the anterior segment parameters after cataract surgery in pseudoexfoliation syndrome (PEX) and control eyes. We conducted a prospective comparative study of 36 eyes (PEX group), 16 eyes (PEXG group) and 46 eyes (control group) of 98 patients after phacoemulsification with intraocular lens implantation. Before surgery, 1 week, 1 month and 3 months postoperatively, anterior chamber parameters were evaluated by swept source anterior segment optical coherence tomography (AS-OCT). Anterior chamber depth (ACD), angle opening distance (AOD500/750), trabecular-iris space area (TISA500/750), trabecular-iris angle (TIA500/750) and lens vault (LV) were assessed at each study visit. Preoperatively, ACD, AOD500/750 and TISA500/750 were significantly smaller, while LV was significantly greater in PEX and PEXG eyes than in controls. 3 months postoperatively all irido-corneal parameters and ACD were significantly greater in all study groups without intergroup differences. ACD and LV significantly increased in PEX group between 1 and 3 months after surgery while being stable in control group. Relative increases in ACD, AOD500, TISA750, TIA500/750 were significantly higher in PEX and PEXG groups than in controls. Our study finds that ACD and iridocorneal parameters in AS-OCT demonstrated significantly greater relative increases 3 months after phacoemulsification in PEX and PEXG groups than in control eyes. Significantly greater deepening of anterior chamber and opening of the irido-corneal angle may be a reason for different refractive outcomes and IOP control in patients with PEX and PEXG after routine cataract surgery.

## Introduction

Pseudoexfoliation (PEX) syndrome, first characterized by Lindberg in 1917^1^, is considered a risk factor for cataract^2^, ocular hypertension and open-angle glaucoma^3^. Although, eyes with PEX have usually open configuration of irido-corneal angle, narrow angles are significantly more frequent in PEX eyes than in general population^4,5^.

Patients with PEX syndrome have a greater long-term IOP decrease after cataract surgery^6–8^. Gonioscopic evaluation of PEX eyes has revealed that 32% of PEX patients had narrow angles compared to the 4% in general population^4,9^. It is well known that in PEX the risk of zonular instability is high and it may lead to refractive surprises and refractive outcomes after cataract surgery are statistically significantly worse in PEX than in healthy eyes^10–12^. Spikes of IOP and progression of glaucoma are common postoperative complications after cataract surgery in PEX eyes.

Anterior segment optical coherence tomography (AS-OCT) is an objective platform to visualise the anterior segment of the eye with high resolution. It can be used for non-invasive quantitative analysis of morphology of the anterior chamber and irido-corneal angle^13–16^.

Ultrasound biomicroscopic (UBM) studies on the anterior segment of PEX eyes have shown abnormalities of the zonules, lens thickening, shallow anterior chamber depth (ACD), and narrow irido-corneal angles^17,18^. AS-OCT is a precise non-contact imaging technique to provide cross-sectional images of the anterior chamber^19^. It can be used for quantitative analysis of anterior segment and irido-corneal angle. Previous studies have already compared cataract surgery in PEX and control eyes^20–23^. However, in this study, we objectively evaluated changes in anterior chamber parameters measured with AS-OCT after cataract surgery in PEX and compared them to healthy eyes. Relatively greater changes in AS-OCT parameters may have an impact on effective lens position (ELP) and may be a reason for refractive surprises after cataract surgery and spikes of IOP in PEX eyes.

## Materials and methods

### Study design

This was a prospective study of 98 eyes of 98 patients with cataract with or without PEX who underwent uncomplicated phacoemulsification surgery with intraocular lens (IOL) implantation. The study was based in the Department of Diagnostics and Microsurgery of Glaucoma of Medical University of Lublin, Poland and the surgeries were performed between October 2016 and November 2017. Written informed consent was obtained from all the participants in accordance with tenets of Declaration of Helsinki. The protocol of this study was approved by the Bioethics Committee of Medical University in Lublin (Poland) and registered under the number KE-0254/241/2014. All methods were carried out in accordance with relevant guidelines and regulations.

Of the 98 eyes, 36 eyes were in the PEX group, 16 eyes in the PEXG (pseudoexfoliation glaucoma) group and 46 eyes in control group and the follow up was 3 months.

### Inclusion and exclusion criteria

The inclusion criteria of the study were patients aged over 18 years with senile cataract only or senile cataract coexisting with PEX or pseudoexfoliation glaucoma (PEXG) with medically controlled intraocular pressure (IOP).

Exclusion criteria included previous eye surgery, corneal pathologies or subjects with other ocular pathologies other than cataract and glaucoma (e.g., inflammatory eye diseases, proliferative diabetic retinopathy, exudative age-related macular degeneration (AMD)), intraoperative complications. We also excluded eyes with manifest iridophacodonesis, intraoperative complications and those in which a capsular tension ring was inserted.

### Surgical procedure

All surgeries were performed using Infiniti (Alcon laboratories, Inc., Fort Worth, TX, USA) under topical anaesthesia by one surgeon (TŻ) through 2.2 mm incision in the upper corneal limbus. The implantation of Aspira-aA (HumanOptics AG, Erlangen, Germany) intraocular lens to the capsular bag was performed in all cases.

### Study protocol

All patients underwent a baseline examination which included assessment of best corrected visual acuity (BCVA), biomicroscopy and measurement of IOP (Goldmann applanation tonometry). The ocular axial length was measured by an optical biometry device (Version V.7.7 IOL Master 500, Carl Zeiss Meditec AG, Jena, Germany) in all patients at a baseline visit.

After surgery, follow-up visits were arranged at 1 week, 1 and 3 months after surgery. At each postoperative visit all the above-mentioned examinations, except biometry were performed.

### Anterior segment optical coherence tomography

Evaluation of the anterior segment parameters was performed with anterior segment swept-source optical coherence tomography (AS-OCT, Casia SS-1000, Tomey, Nagoya, Japan). The Casia SS-1000 is a swept-source Fourier-domain AS-OCT that uses a scanning light source of 1310 nm wavelength with a measuring speed of 30 000 axial scans per second. The axial resolution is 10 μm and transverse resolution is 30 μm. The system performs large depth scans (6 mm tissue penetration) with dimensions of 16 mm (length) and 16 mm (width).

An experienced operator (AK) performed and analysed all scans at a baseline examination before the surgery, at 1 week, 1 month and 3 months after surgery. All anterior segment parameters were measured before mydriasis in dark room. The scan was centered on the pupil and the morphology of structures on the nasal side of the eye were analysed with the built-in software. Data were excluded if the scleral spur could not be identified.

The imaging was performed in Anterior Segment and Angle Analysis mode with correct head position. The parameters that were used to quantify the anterior segment of the eye were as follows:

1. Angle opening distance at 500 μm and 750 μm (AOD500 and AOD750, respectively): the distance between posterior corneal surface and the anterior iris surface on a line perpendicular to the trabecular meshwork, measured at 500 μm and 750 μm from scleral spur, respectively^24^.
2. Trabecular-iris space area at 500 μm and 750 μm (TISA500 and TISA750, respectively): the trapezoidal area with the following boundaries: anteriorly, AOD500 and AOD750, respectively; posteriorly a line drawn from the scleral spur perpendicular to the plane of the inner scleral wall to the iris; superiorly, the inner corneoscleral wall, and inferiorly, the iris surface^14^.
3. Trabecular-iris angle at 500 μm and 750 μm (TIA500 and TIA750, respectively): angle with the apex in the iris recess and the arms of the angle passing through a point on the trabecular meshwork 500 μm and 750 μm, respectively, from the scleral spur and the point on the iris perpendicularly opposite^24^.
4. Anterior chamber depth (ACD): defined as a distance from the corneal endothelium to the anterior surface of the lens.
5. Lens vault (LV): The perpendicular distance between the anterior pole of the lens and the horizontal line connecting the two scleral spurs.
6. Pupil diameter (PD): was measured along the horizontal (temporal-nasal) meridian.

### Power and sample size calculation

To detect a difference of one standard deviation of ACD, assuming a standard deviation (SD) of 0.3 mm a two-sided α error of 5% and achieving a power of 80%, the sample size of 42 eyes (14 eyes in each group) was calculated.

### Statistical analysis

The statistical analysis was performed using SPSS for Mac (version 26.0.0.1; IBM SPSS Statistics, Chicago, IL, USA). Differences were considered statistically significant at *p* < 0.05. Normality of data was assessed using Kolmogorov–Smirnov test. Categorical variables were analysed using the Chi-square (χ^2^) test. For continuous variables between the study groups, analysis of variance (ANOVA) and Kruskal–Wallis test were used for analysis. Within-group comparisons of continuous variables were performed using the Wilcoxon signed-rank test. To compare the anterior segment parameters between study groups, ANOVA was used. To compare changes of anterior segment parameters after surgery within certain study group, repeated measures analysis of variance (RM-ANOVA) with a Greenhouse–Geisser correction was used. The Bonferroni correction was applied for multiple comparisons. Correlation between different parameters was studied by Spearman rank correlation.

## Results

36 eyes (PEX group), 16 eyes (PEX group) and 46 eyes (control group) of 98 patients were enrolled to the study. All patients completed the follow-up schedule of 3 months and were analysed. No postoperative complications i.e., posterior capsule opacification (PCO), IOL decentration or tilt were observed during the follow-up.

The patients’ demographics are shown in Table 1. No statistically significant differences between the groups in the age, sex, right/left eye, central corneal thickness, preoperative best corrected visual acuity, mean axial length, mean corneal power and mean IOL power were noted (*p* > 0.05). The mean preoperative IOP was statistically significantly higher in PEXG group (*p* = 0.049). Post-hoc analysis with Dunn-Bonferroni pairwise test was conducted, resulting with a significance level at 0.031 between control and PEXG eyes. There was no significant difference in IOP between PEX and PEXG eyes and control and PEX eyes (*p* = 0.238, *p* = 1.000; respectively).

**Table 1:** Baseline characteristics.

Characteristic	Mean ± SD			*p* value
	Control *n* = 46	PEX *n* = 36	PEXG *n* = 16	
Age (y)	76.11 ± 10.44	80.03 ± 5.42	77.56 ± 5.51	0.208^a^
Sex (male/female)	22 / 24	15 / 21	7 / 9	0.852^b^
Right/Left Eye	22 / 24	17 / 19	9 / 7	0.816^b^
AxL (mm)	23.44 ± 0.91	23.40 ± 0.76	23.72 ± 0.59	0.156^c^
K (D)	43.50 ± 1.40	43.64 ± 1.17	43.40 ± 1.30	0.890^c^
PD (mm)	3.27 ± 0.91	3.28 ± 0.75	3.25 ± 0.82	0.995^a^
IOL power (D)	21.42 ± 2.19	21.35 ± 1.79	20.78 ± 0.71	0.307^c^
IOP (mmHg)	15.00 ± 2.95	15.89 ± 3.42	18.69 ± 5.34	0.049^c^*
BCVA (Snellen)	0.34 ± 0.25	0.36 ± 0.22	0.32 ± 0.22	0.949^c^
CCT (μm)	539.54 ± 31.22	520.42 ± 37.23	535.44 ± 41.62	0.075^c^

^a^Analysis of variance (ANOVA); ^b^Chi-square (χ^2^) test; ^c^Kruskal-Wallis test.

AxL = axial length; K = keratometry; IOL = intraocular lens; y = year; mm = millimeter; D = diopter; CCT = central corneal thickness.

### Anterior chamber and angle parameters before surgery

The anterior segment parameters measured by AS-OCT on preoperative visits are summarized in Table 2.

**Table 2:** Pre- and postoperative anterior segment parameters in study groups.

			Postoperative					
Parameter	Group	Preoperative (I)	1 month (II)	3 months (III)	*p*^*2*^	*p* I vs II	*p* I vs III	*p* II vs II
ACD (mm)	Control	2.74 ± 0.33	3.60 ± 0.34	3.66 ± 0.32	****	****	****	NS
	PEX	2.47 ± 0.41	3.45 ± 0.32	3.62 ± 0.30	****	****	****	*
	PEXG	2.40 ± 0.40	3.30 ± 0.37	3.46 ± 0.46	****	****	****	NS
	*p*^*1*^	***	**	NS	****	****	****	
AOD500 (mm)	Control	0.44 ± 0.20	0.66 ± 0.19	0.65 ± 0.16	****	****	****	NS
	PEX	0.33 ± 0.13	0.60 ± 0.15	0.60 ± 0.13	****	****	****	NS
	PEXG	0.30 ± 0.11	0.55 ± 0.13	0.58 ± 0.15	****	****	****	NS
	*p*^*1*^	**	*	NS	****	****	****	NS
AOD750 (mm)	Control	0.60 ± 0.25	0.94 ± 0.23	0.93 ± 0.20	****	****	****	NS
	PEX	0.47 ± 0.18	0.87 ± 0.21	0.87 ± 0.18	****	****	****	NS
	PEXG	0.42 ± 0.15	0.81 ± 0.19	0.84 ± 0.21	****	****	****	NS
	*p*^*1*^	**	NS	NS	****	****	****	NS
TISA500 (mm^2^)	Control	0.17 ± 0.08	0.24 ± 0.07	0.23 ± 0.06	****	****	****	NS
	PEX	0.13 ± 0.04	0.21 ± 0.05	0.21 ± 0.05	****	****	****	NS
	PEXG	0.12 ± 0.03	0.18 ± 0.04	0.20 ± 0.05	****	****	****	NS
	*p*^*1*^	**	*	NS	****	****	****	NS
TISA750 (mm^2^)	Control	0.30 ± 0.13	0.44 ± 0.12	0.43 ± 0.11	****	****	****	NS
	PEX	0.23 ± 0.08	0.40 ± 0.10	0.39 ± 0.08	****	****	****	NS
	PEXG	0.21 ± 0.06	0.36 ± 0.08	0.38 ± 0.10	****	****	****	NS
	*p*^*1*^	**	*	NS	****	****	****	NS
TIA500 (degree)	Control	34.16 ± 9.41	47.61 ± 7.30	47.16 ± 5.97	****	****	****	NS
	PEX	31.02 ± 9.73	48.86 ± 6.77	49.45 ± 6.10	****	****	****	NS
	PEXG	28.80 ± 9.25	45.99 ± 7.95	47.56 ± 9.11	****	****	****	NS
	*p*^*1*^	NS	NS	NS	****	****	****	NS
TIA750 (degree)	Control	33.44 ± 9.18	47.66 ± 6.88	47.32 ± 5.50	****	****	****	NS
	PEX	30.15 ± 9.08	48.10 ± 6.91	48.52 ± 6.27	****	****	****	NS
	PEXG	27.63 ± 9.07	45.53 ± 8.23	47.00 ± 8.78	****	****	****	NS
	*p *^*1*^	NS	NS	NS	****	****	****	NS
LV (mm)	Control	0.39 ± 0.25	−0.45 ± 0.42	−0.51 ± 0.35	****	****	****	NS
	PEX	0.56 ± 0.28	−0.24 ± 0.35	−0.31 ± 0.31	****	****	****	NS
	PEXG	0.64 ± 0.27	−0.26 ± 0.46	−0.38 ± 0.43	****	****	****	NS
	*p *^*1*^	***	*	*				

NS = non significant; *p ≤ 0.05; **p ≤ 0.01; ***p ≤ 0.001; ****p ≤ 0.0001.

^1^Analysis of variance (ANOVA).

^2^Repeated measures analysis of variance (RM-ANOVA) with a Greenhouse–Geisser correction; post hoc analysis with a Bonferroni adjustment.

ACD was statistically significantly shallower in PEX and PEXG eyes than in controls. Post-hoc analysis with Tukey’s test did not reveal differences between PEX and PEXG eyes.

All angle parameters except of TIA500 and TIA750 displayed significant differences between study groups. AOD500, AOD750, TISA500 and TISA750 were statistically significantly smaller in both PEX and PEXG eyes than in controls. LV was statistically significantly higher in both PEX and PEXG group than in controls. Post-hoc analysis with Tukey’s test did not reveal differences between PEX and PEXG eyes in any of there parameters.

### Anterior chamber and angle parameters after surgery

The anterior segment parameters measured by AS-OCT on postoperative visits are summarized in Table 2.

During the postoperative period all the parameters of anterior chamber and iridocorneal angle have significantly increased compared to the baseline values in all study groups. There was a significant difference between all the AS-OCT parameters between preoperative and both 1 and 3 months postoperative values (p < 0.0001 in all cases).

**Figure 1:**
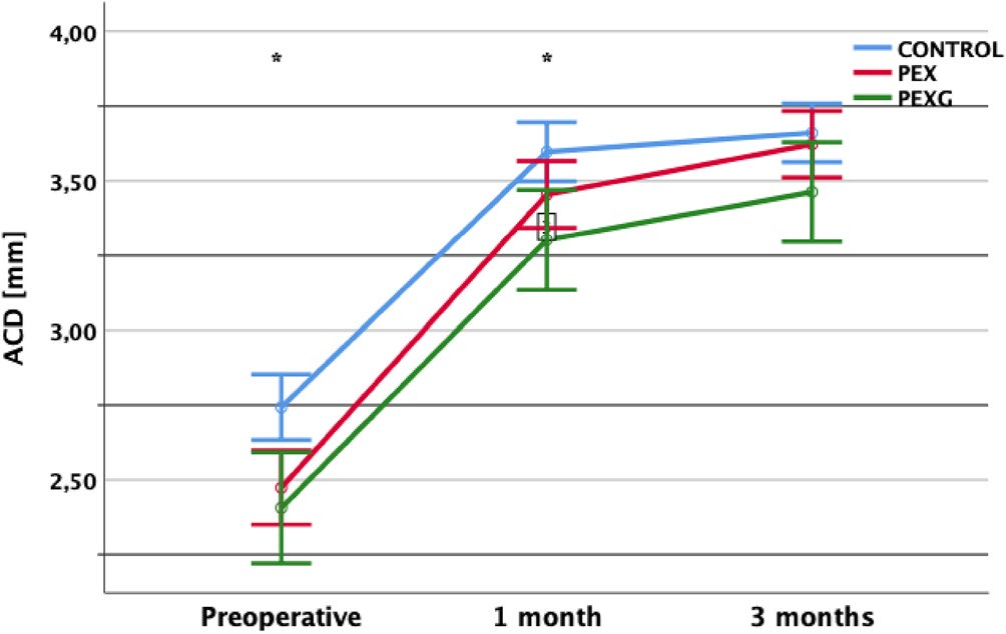
AS-OCT assessment of ACD before and after phacoemulsification in PEX, PEXG and control eyes. There were statistically significant differences in ACD in 3 study groups preoperatively and 1 months after surgery. *p < 0.05 Control versus PEX versus PEXG group (ANOVA); PEX = pseudoexfoliation syndrome; PEXG = pseudoexfoliation glaucoma; ACD = anterior chamber depth.

ACD was statistically significantly shallower in PEX and PEXG eyes than in controls 1 month postoperatively. Post-hoc analysis with Tukey’s test did not reveal differences between PEX and PEXG eyes. 3 months after the surgery, ACD did not differ statistically significantly before study groups (Fig. 1). Moreover, there was a significant difference between ACD 1 and 3 months in PEX eyes with no difference in control group.

LV which was significantly higher in PEX and PEXG eyes preoperatively, significantly changed after surgery in all study groups. Interestingly, after surgery there were still significant changes in postoperative LV between study groups (Table 2).

All angle parameters except of AOD750, TIA500 and TIA750 displayed significant differences between study groups 1 month postoperatively. AOD500, TISA500 and TISA750 were statistically significantly smaller in both PEX and PEXG eyes than in controls. Post-hoc analysis with Tukey’s test did not reveal differences between PEX and PEXG eyes.

**Figure 2:**
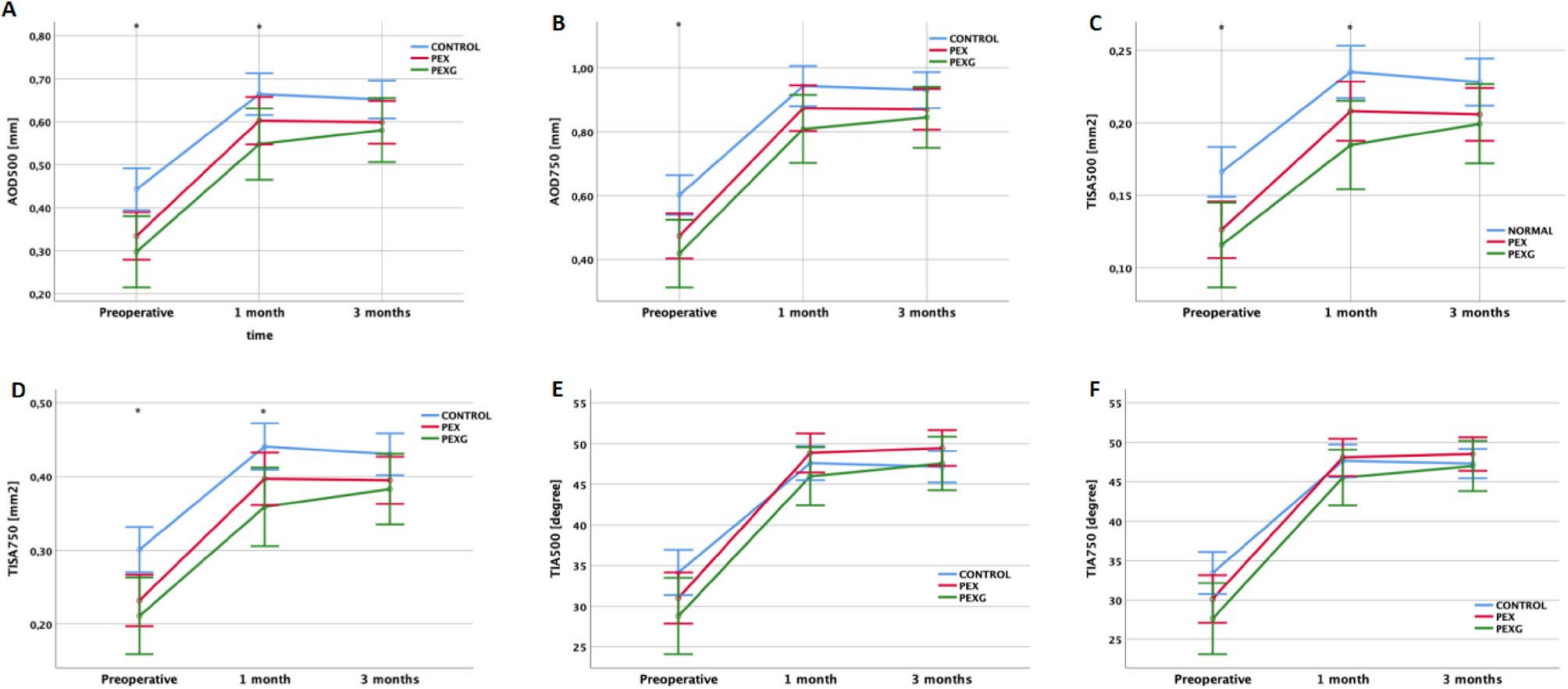
AS-OCT assessment of irido-corneal angle parameters before and after phacoemulsification in PEX, PEXG and control eyes. (**A, C, D**) There was statistically significant difference between between 3 study groups preoperatively and 1 month after surgery in terms of AOD500, TISA500 and TISA750, respectively; (**B**) There was a statistically significant difference between 3 study groups in terms of AOD750; (**E, F**) There were no statistically significant differences between 3 study groups in terms of TIA500 and TIA750, respectively. *p < 0.05 Control versus PEX versus PEXG group (ANOVA); PEX = pseudoexfoliation syndrome; PEXG = pseudoexfoliation glaucoma; AOD500 = Angle opening distance at 500 μm; AOD750 = Angle opening distance at 750 μm; TISA500 = Trabecular-iris space area at 500 μm; TISA750 = Trabecular-iris space area at 750 μm; TIA500 = Trabecular-iris angle at 500 μm; TIA750 = Trabecular-iris angle at 750 μm.

Although there was no significant difference between the groups, all parameters except of TIA500 and TIA750 were higher in control than PEX and PEXG groups 3 months postoperatively (Fig. 2).

Postoperatively, all study groups displayed statistically significant IOP reduction (*p* ≤ 0.001 for all groups, Wilcoxon signed-rank test). After surgery IOP in control group decreased to 13.61 mmHg ± 2.72, in PEX group to 14.78 mmHg ± 3.22 and in PEXG group to 16.44 mmHg ± 4.60. The mean postoperative IOP was statistically significantly higher in PEXG group (*p* = 0.012, ANOVA). Post-hoc analysis with Dunn-Bonferroni pairwise test was conducted, resulting with a significance level at 0.010 between control and PEXG eyes. There was no significant difference in IOP between PEX and PEXG eyes and control and PEX eyes (*p* = 0.214, *p* = 0.247; respectively).

Mean increases of all AS-OCT parameters were significantly higher in PEX and PEXG groups than in control groups except of LV, AOD750 and TISA 500, where difference was higher, without statistical significance. Post-hoc analysis with Dunn-Bonferroni pairwise test revealed that no significant differences were observed in changes of all parameters between PEX and PEXG group (Table 3). LV changes were similar in all study groups. However, percentage relative changes were lower in PEX and PEXG group than in controls.

**Table 3:** Changes in anterior chamber parameters before and 3 months after phacoemulsification in three study groups.

Change of parameter	Mean ± SD (% change)			*p* value
	Control *n* = 46	PEX *n* = 36	PEXG *n* = 16	
ACD (mm)	0.92 ± 0.39 (33.6%)	1.15 ± 0.39 (46.6%)	1.06 ± 0.44 (44.2%)	0.036*
AOD500	0.21 ± 0.13 (47.7%)	0.26 ± 0.12 (78.8%)	0.28 ± 0.09 (93.3%)	0.042*
AOD750	0.33 ± 0.18 (55.0%)	0.40 ± 0.16 (85.1%)	0.43 ± 0.14 (102.4%)	0.066
TISA500	0.06 ± 0.04 (35.3%)	0.08 ± 0.04 (61.5%)	0.08 ± 0.03 (66.7%)	0.062
TISA750	0.13 ± 0.08 (43.3%)	0.16 ± 0.07 (69.6%)	0.17 ± 0.06 (81.0%)	0.048*
TIA500	13.00 ± 7.43 (38.1%)	18.43 ± 7.71 (59.4%)	18.77 ± 6.13 (65.2%)	0.002*
TIA750	13.87 ± 7.61 (41.5%)	18.38 ± 6.45 (61.0%)	19.37 ± 6.16 (70.1%)	0.004*
LV	0.90 ± 0.40 (335.2%)	0.87 ± 0.38 (183.0%)	1.03 ± 0.44 (174.0%)	0.444

Analysis of variance (ANOVA).

The correlation between preoperative anterior chamber parameters and percentage change in IOP 3 months after surgery was analysed. No statistically significant correlation was found in neither in control group nor in PEXG group. Interestingly, in PEX group weak negative correlation was found between AOD500, AOD750, TISA500, TISA750, TIA750 and percentage change in IOP 3 months postoperatively (Table 4).

**Table 4:** Correlation between preoperative angle parameters and percentage change in IOP 3 months postoperatively in PEX group.

Preoperative parameter	% Change in IOP	*p* value
	Spearman’s rank correlation coefficient	
ACD	– 0.202	0.237
AOD500	– 0.339*	0.043*
AOD750	– 0.345*	0.039*
TISA500	– 0.383*	0.021*
TISA750	– 0.333*	0.047*
TIA500	– 0.313	0.063
TIA750	– 0.340*	0.042*
LV	0.076	0.660

## Discussion

The aim of this study was to objectively analyze changes in anterior segment configuration after routine uncomplicated cataract surgery in PEX, PEXG and control eyes. It confirms that there is a significant increase in anterior chamber parameters after routine cataract surgery and what is more interesting, that this increase is much more pronounced in PEX and PEXG eyes. It is well known that change of these parameters may lead to spikes of IOP and refractive surprises with hyperopic shift and in our study we quantitatively evaluated changes of anterior segment parameters in PEX, PEXG and control eyes.

In our study, mean preoperative ACD, AOD500, AOD750, TISA500 and TISA750 were significantly lower in PEX and PEXG eyes than in controls. Both TIA500 and TIA750 were also lower in PEX and PEXG eyes but these differences were not statistically significant. These results are consistent with the results achieved by others. Doganay et al.^25^ found that ACD in PEX glaucoma eyes was significantly lower than in control group. In a study by Gharagozloo et al.^26^ anterior chamber volumes were significantly smaller in both affected and unaffected eyes with unilateral PEX compared with control eyes. Gur Gungor et al.^27^ showed that mean preoperative ACD values in PEX group (3.04 ± 0.5 mm) were lower than the normal group (3.26 ± 0.3 mm) but the difference was not statistically significant. Elgin et al.^28^ found that PEX eyes have relatively shallower anterior chambers than control eyes. Shallow ACD in eyes with PEX and PEXG may be related to higher LV. This finding was consistent with other study where LV was measured in PEX and control eyes^9^. LV which is considered to be independent ocular parameter associated with angle closure, was significantly higher both in PEX and PEXG group^29^.

However, whereas anterior chamber depth in PEX and healthy eyes was extensively studied, much less research has been done to evaluate other anterior chamber and irido-corneal angle parameters in AS-OCT. Zheng et al.^30^ found that differences in anterior chamber angle parameters, AOD500, TISA500 and TIA500 were significantly smaller in PEX eyes than in their fellow eyes or normal controls. Interestingly, these differences were observed only when the pupil was constricted by light, being not significant in the dark. Also Mohammadi et al.^9^ found that anterior chamber angle parameters (AOD500, AOD750, TISA500 and TISA750) were significantly lower in PEXG group compared with PEX and control eyes. In this study, all the AS-OCT parameters were similar to those obtained by us preoperatively. Recently, Kassos et al.^31^ found that PEX, compared with non-PEX eyes, demonstrated a greater ACD deepening and a hyperopic shift after phacoemulsification. Similarly to our study, the authors did not find statistically significant differences between the two groups in terms of ACD and irido-corneal angle parameters postoperatively. However, they also found that percentile increases of ACD was significantly higher in PEX eyes compared with non-PEX eyes.

There is a growing body of evidence that anterior shift of the lens, secondary to the subclinical weakness of the zonular fibers caused by accumulation of the pseudoexfoliative material, may play a role in the development of PEXG in PEX eyes^9,32,33^. According to our observations, preoperative angle parameters had significant negative correlation with percentage of change in postoperative IOP in PEX eyes. Interestingly, all these parameters were lower in PEXG than in PEX eyes, without statistical significance. This is consistent with other studies, where the authors raise the issue of the role of angle-closure mechanisms in patomechanisms of PEXG^9^. It also emphasizes the importance of isolating PEXG as a separate group to identify factors that may have a role in progression from PEX syndrome to PEXG.

Several studies demonstrated that cataract surgery can deepen anterior chamber and increase the width of irido-corneal angle^27,34,35^. However, our study provides insight into the rate of changes in AS-OCT parameters after phacoemulsification in PEX eyes which is much higher than in healthy eyes. It can explain the theory that cataract extraction may be protective against the development of glaucoma, especially in PEX eyes^36^. Moreover, relatively greater changes in AS-OCT parameters may have an impact on effective lens position (ELP) and may be a reason for refractive surprises after cataract surgery in PEX eyes. In our previous study^10^ we analysed refractive outcomes after cataract surgery in PEX and control eyes. We found statistically significantly higher mean absolute errors and lower percentages of eyes within ± 0.5 in PEX eyes than in controls. It may be related to large LV and shallow ACD in PEX, resulting in a more posterior IOL position than expected and hyperopic shift. Moreover, larger LV is often accompanied with changes in zonular laxity which may lead to deeper postoperative ACD^29,37^. Fallah Tafti et al.^12^ measured the pseudophakic ACD change after cataract surgery in patients with PEX using optical coherence tomography (OCT) Visante. They found a significant increase in postoperative ACD from 1 to 6 months after cataract surgery and associated this backward movement of the IOL with concurrent refractive errors. These findings are consistent with our results. There was statistically significant increase in postoperative ACD between 1 and 3 months postoperatively in PEX eyes, whereas postoperative ACD in control eyes remained stable between 1 and 3 months. Similarly in PEXG eyes, postoperative ACD changed from 3.30 ± 0.37 in 1 month to 3.46 ± 0.46 in 3 months, but this difference was not statistically significant, probably because of small sample size of the PEXG group. This may have a clinical impact on stabilization of refractive outcomes in PEX eyes which is longer than in healthy eyes.

Strengths of the current study are the use of homogenous group of eyes with separation of PEXG group, implantation of a single IOL model, surgery performed by a single surgeon.

There are several limitations to this study. First, the relatively small sample size, especially in PEXG group. Second, lack of analysis of irido-corneal angle in AS-OCT in light illumination, which may give additional information about pathophysiology of glaucoma.

In conclusion, in this prospective comparative study, we demonstrated statistically significant differences in changes in anterior chamber and irido-corneal angle parameters in AS-OCT between control eyes and PEX and PEXG eyes after phacoemulsification. These changes may be a reason for different refractive outcomes and IOP control in patients with PEX and PEXG after routine cataract surgery. Finally, the preoperative ACD in control group is slightly smaller than in average population.

## Data Availability

Data are available on reasonable request from Aleksandra Wlaź (aleksandra.wlaz@icloud.com).

## Abbreviations

AS-OCT: Anterior segment–optical coherence tomography
PEX: Pseudoexfoliation syndrome
PEXG: Pseudoexfoliation glaucoma
IOP: Intraocular pressure
BCVA: Best corrected visual acuity
SD: Standard deviation
ANOVA: Analysis of variance
